# Dor Precordial em Idoso e Infarto. Não é Tão Elementar, Meu Caro Watson!

**DOI:** 10.36660/abc.20210331

**Published:** 2021-06-08

**Authors:** Ricardo Wang, José Carlos da Costa Zanon, Fernando Carvalho Neuschwander

**Affiliations:** 1 Instituto Orizonti Belo HorizonteMG Brasil Instituto Orizonti , Belo Horizonte , MG - Brasil; 2 Hospital UNIMEDBH Belo HorizonteMG Brasil Hospital UNIMEDBH , Belo Horizonte , MG - Brasil; 3 Universidade Federal de Ouro Preto Ouro PretoMG Brasil Universidade Federal de Ouro Preto , Ouro Preto , MG - Brasil

**Keywords:** Dor no Peito, Angina Pectoris, Idoso, Envelhecimento, Infarto do Miocárdio/diagnóstico, Síndrome Coronariana Aguda/diagnóstico

Aprendemos na faculdade de medicina o modo tradicional de fazer processo investigativo, para chegar ao diagnóstico. O processo é classicamente baseado na coleta da anamnese e exame clínico, raciocínio clínico, geração de hipótese, e teste com exames complementares. Em geral, seguimos o princípio da Navalha de Ockham: “a resposta mais simples é em geral a correta”, ou como dizia William Osler: “Onde se escuta cascos, não pense em zebras”. Mas nesse processo investigativo, o raciocínio clínico é desafiado numa população idosa acima dos 80 anos de idade, e nem sempre a resposta mais simples é a mais correta. Nessa faixa etária, é comum a presença de várias comorbidades, que podem interferir, alterando a percepção dos sintomas cardiovasculares (depressão, demência, medicamentos que interferem no sistema nervoso central, diabetes, analgésicos, etc.), ou modificar a sintomatologia. A presença de sintomas atípicos, como dispneia, sudorese, vômitos, diarreia, dor epigástrica, e confusão mental, pode mascarar a patologia cardiovascular. ^1 , 2^

Vários estudos demonstram a dificuldade de diagnosticar o infarto agudo do miocárdio (IAM) nessa população, o que, logicamente, acarreta atraso ou subtratamento. Mesmo quando estabelecidos o diagnóstico e o tratamento precoce do IAM, a mortalidade permanece alta nessa população. O subtratamento ou falta de tratamento contribuem para o aumento da mortalidade nessa faixa etária. ^2 - 4^ O entendimento de fatores que dificultam o diagnóstico é de suma importância para estabelecermos protocolos direcionados para os muito idosos.

Era de se esperar que os médicos aplicassem o método analítico e metodologia no processo investigativo, mas não é o que ocorre na prática. Há outros fatores que influenciam o processo de diagnóstico. Dentre eles, fatores cognitivos, inicialmente descritos por Kahneman e Tversky ^5^ (ganhador do prémio Nobel de economia), são descritos como atalhos mentais, intuitivos, para chegarmos rapidamente ao resultado (diagnóstico). Esse método é subjetivo e muito influenciado por fatores emocionais, e tem alta chance de erros. ^6^ Para pacientes que se apresentam no serviço de emergência com dor precordial, é até intuitivo descartar IAM. Aplicando protocolos bem estabelecidos para pacientes com dor precordial, temos alta acurácia diagnóstica para Síndrome Coronariana Aguda. Porém, a presença de sintomatologia atípica abre um leque de possibilidades diagnósticas, ^7^ e há necessidade de um atendimento sistematizado para o diagnóstico correto. A aplicação desses atalhos mentais, seja ela decorrente de urgência, sobrecarga de trabalho (comum no setor da emergência), estresse, ou lacunas na formação e capacitação no atendimento, leva frequentemente à diminuição da acurácia diagnóstica.

No elegante estudo conduzido pelo Filgueiras et al., ^8^ destaca-se a necessidade de 6 anos de coleta, para se obter uma amostra adequada de idosos acima de 80 anos (o que demonstra a dificuldade de estudar esse subgrupo populacional). O estudo foi conduzido num centro terciário (unidade coronariana - UCO), onde os pacientes foram previamente triados na emergência, setor onde ocorre a maioria dos equívocos diagnósticos. Podemos observar, nos dados apresentados, que a população idosa tinha 83% de troponina positiva, e 41% de pacientes com lesões obstrutivas vistas na coronariografia, sugerindo que os idosos com sintomas típicos, e associados com alteração do marcador de necrose miocárdica, tiveram maior chance de serem admitidos na UCO. Nessa amostra, o viés de seleção pode constituir um problema para validade externa dos dados obtidos.

Digna de nota é a falta de menção à descrição das comorbidades, além de um tema muito relevante que é o grau de fragilidade da população estudada. É muito comum os médicos não instituírem o tratamento completo para IAM, seja devido à baixa expectativa de vida, ou pelo risco de complicações, tais como sangramento. Portanto, esse grupo também tem menor chance de ser encaminhado para a UCO.

Nesse estudo, apesar de os pacientes muito idosos apresentarem sintomas típicos, quando comparados a pacientes mais jovens (índice de tipicidade 3,41 ± 1,77 x 3,49 ± 1,77; p=0,61), o resultado se contrapõe aos achados do estudo de Brieger, ^4^ em que a prevalência de sintomas atípicos foi de 14% em pacientes acima de 75anos, e somente 5% se < 65 anos. Dos pacientes com sintomas atípicos, somente 60% receberam corretamente o diagnóstico de SCA ^4^ . Ademais, a análise dos sintomas atípicos seria fundamental, para entender fatores que contribuem para a dificuldade diagnóstica e as chaves para o diagnóstico. Além do estudo dos sintomas, outro aspecto fundamental é a análise do processo investigativo médico: forma de coleta das informações, coleta de informações com cuidadores, tempo dispendido, forma de processamento das informações, e métodos complementares utilizados.

Em resumo, apesar de a maioria dos pacientes apresentar sintomas típicos, associados ao aumento de marcadores de necrose miocárdica, pacientes muito idosos, com sintomas atípicos, ainda representam um desafio ao médico, e, nessas condições, a arte médica investigativa e sistemática deve ser aplicada. Além disso, ainda carecemos de protocolos específicos para essa população.
