# Supplementary material for: Dor Precordial em Idoso e Infarto. Não é Tão Elementar, Meu Caro Watson!
Source: Arq Bras Cardiol. 2021 Jun 8;116(6):1046–7. [Article in Portuguese] doi: 10.36660/abc.20210331 (PMC8288537; doi:10.36660/abc.20210331)
Supplement: Translation

# Translation: Precordial Pain and Infarction in the Elderly. It´S no so Elementary, My Dear Watson!

**Authors:** Ricardo Wang, José Carlos da Costa Zanon, Fernando Carvalho Neuschwander

**Affiliations:** 1 Brazil Instituto Orizonti, Belo Horizonte, MG - Brazil; 2 Brazil Hospital UNIMEDBH, Belo Horizonte, MG - Brazil; 3 Brazil Universidade Federal de Ouro Preto, Ouro Preto, MG - Brazil

**Keywords:** Chest Pain, Angina Pectoris, Aged, Aging, Myocardial Infarction/diagnosis, Acute Coronary Syndrome/diagnosis

In Medical School, we learn the traditional way to conduct the investigative process in order to reach a diagnosis. This process is traditionally based on the collection of anamnesis and clinical exams, clinical reasoning, the generation of a hypothesis, and tests with complementary exams. In general, we follow the the Occam’s razor principle: “the simplest answer is usually the correct answer,” or as William Osler’s aphorism: “Where you hear hoofs, you don´t think of zebras”. However, in this investigative process, the clinical reasoning is challenged in an elderly population over 80 years of age, and not always is the simplest answer the most correct. In this age group, the presence of comorbidities is quite common and can interfere, changing the perception of cardiovascular symptoms (depression, dementia, medications that interfere in the central nervous system, diabetes, analgesics, etc.), or even modify the symptomatology. The presence of atypical symptoms, such as dyspnea, sudoresis, vomiting, diarrhea, epigastric pain, and mental confusion can all mask the cardiovascular pathology.^1 , 2^

Many studies have shown the difficulty in diagnosing acute myocardial infarction (AMI) in this population, which clearly entails delays in or the lack of treatment. Even when the diagnosis and early treatment of AMI is established, the mortality rate remains high in this population. The under-treatment or lack of treatment contribute to the increase in mortality in this age group.^2 - 4^ Understanding the factors that make the diagnosis difficult is of utmost importance in establishing protocols directed toward the older age elderly patient.

It is expected that doctors will apply the analytical method and methodology in the investigative process, but this not what happens in practice. There are other factors that influence the diagnostic process. Among these are cognitive factors, first described by Kahneman and Tversky^5^ (winner of the Nobel Prize for economy), which are described as mental, intuitive shortcuts to reach the result (diagnosis) quickly. This method is subjective, is highly influenced by emotional factors, and has a high risk for error.^6^ For patients who receive medical care at emergency clinics complaining of precordial pain, it is even intuitive to discard AMI. Applying well-established protocols for patients with precordial pain, we can attain a high diagnostic accuracy for Acute Coronary Syndrome (ACS). However, the presence of atypical symptomatology opens the door to diagnostic possibilities,^7^ and there is the need for a systematized care to reach the proper diagnosis. The application of these mental shortcuts, be they due to urgency, work overload (common in the emergency care sector), stress, or gaps in the formation and training in medical care embracement, often leads to a decline in diagnostic accuracy.

In the impressive study conducted by Filgueiras et al.,^8^ what stands out is the need for a 6-year data collection to obtain an adequate sample of elderly patients over 80 years of age (which shows the difficulty to study this population subgroup). Their study was conducted at a tertiary center (Coronary Care Unit - CCU), where the patients had been previously triaged in the emergency departament, the sector where most incorrect diagnoses occur. It was possible to observe, from the data presented, that 83% of the elderly population presented positive troponin, while 41% presented obstructive lesions observed in the coronarography, suggesting that the elderly patients with typical symptoms, and associated with markers of myocardial necrosis, had a higher chance of being admitted to the CU. In this sample, the selection bias can constitute a problem for the external validity of the obtained data.

It is important to note that the description of the comorbidities was missing, as was another a highly relevant theme, the degree of fragility of the studied population. It is very common for doctors not to apply the complete treatment for AMI, be it due to the low life expectancy or to the risk of complications, such as bleeding. Therefore, this group has a lower chance of being referred to the CU.

In this study, although the very elderly patients presented typical symptoms, when compared to younger patients (typicality index 3.41 ± 1.77 x 3.49 ± 1,77; p=0.61),the result runs in direct contrast with findings from Brieger,^4^ in which the prevalence of atypical symptoms was of 14% in patients over 75, and only 5% if < 65 years of age. Of the patients with atypical symptoms, only 60% received the proper diagnosis of ACS.^4^ Moreover, the analysis of the atypical symptoms would be essential in order to understand factors that contribute to the diagnostic difficulty and the keys to the diagnosis. In addition to the study of the symptoms, another essential aspect is the analysis of the medical investigative process: the manner in which the information is collected, the collection of information with caregiver, the time spent, the manner in which the information is processed, and the complementary methods used.

In sum, although most of the patients presented typical symptoms, associated with an increase in myocardial necrosis markers, very elderly patients with atypical symptoms still represent a challenge for doctors and, under these conditions, the investigative and systematic medical art must be applied. Furthermore, there is still a lack of specific protocols for this population.
